# An Up‐to‐Date Review of Traditional Chinese Medicine in the Treatment of Atherosclerosis: Components, Mechanisms, and Therapeutic Potentials

**DOI:** 10.1002/ptr.70037

**Published:** 2025-07-09

**Authors:** Dilaram Nijat, Qingzhe Zhao, Gulhasal Abdurixit, Jianhua He, Haipeng Liu, Jinyao Li

**Affiliations:** ^1^ School of Pharmaceutical Sciences and Institute of Materia Medica Xinjiang University Urumqi China; ^2^ College of Life Science and Technology, Xinjiang Key Laboratory of Biological Resources and Genetic Engineering Xinjiang University Urumqi China; ^3^ Uygur Medical Hospital of Xinjiang Uygur Autonomous Region Urumqi China; ^4^ Centre for Intelligent Healthcare, Coventry University Coventry UK

**Keywords:** atherosclerosis, natural product, pharmacology, traditional Chinese medicine (TCM)

## Abstract

Atherosclerosis is a chronic inflammatory disease and a major global health concern. In recent years, traditional Chinese medicines (TCMs) have demonstrated multi‐target therapeutic potential against atherosclerosis by modulating inflammatory responses, oxidative stress, lipid metabolism, and gut microbiota interactions. This review systematically evaluates (1) the anti‐atherogenic mechanisms of key TCM herbs (e.g., *Panax ginseng*, *Salvia miltiorrhiza* ) and bioactive compounds (e.g., ginsenosides, tanshinones), (2) their synergistic effects in proprietary formulations, and (3) clinical evidence for cardiovascular protection. Notably, we highlight how TCM compounds like berberine and resveratrol target the gut‐vascular axis by regulating microbiota‐derived metabolites (e.g., TMAO) and improving endothelial function. While preclinical studies show promising results through Nrf2/HO‐1 activation, NF‐κB inhibition, and plaque stabilization, translational challenges persist, including the lack of standardization and microbiome‐dependent efficacy variations. We conclude that integrating TCM's multi‐component advantages with cutting‐edge technologies such as bioinformatics, nanotechnology, and patient‐specific multiomics including microbiome profiling could revolutionize atherosclerosis management, though rigorous clinical validation and standardization remain imperative.

## Graphical abstract

Traditional Chinese medicine in atherosclerosis: multi‐target modulation of pathogenesis.

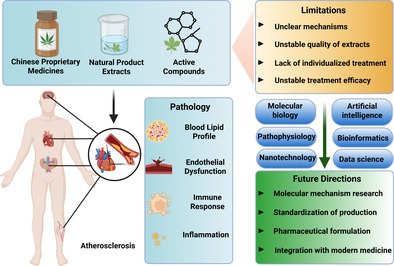

## 1 Introduction

Cardiovascular disease is a leading cause of morbidity and mortality, with atherosclerosis as its primary underlying factor, making it a critical global public health issue (Williams 2024). Atherosclerosis is a chronic cardiovascular condition characterized by fibrofatty lesions in the arterial walls and narrowing of the arterial lumen. Atherosclerosis can affect various tissues and organs throughout the body, leading to several symptoms and clinical events (Matsuoka et al. 2022). In coronary arteries, atherosclerosis can lead to myocardial infarction and heart failure. In intracranial arteries, atherosclerosis plaques can lead to the narrowing or rupture of cerebral blood vessels, potentially causing transient ischemic attacks, ischemic strokes, or hemorrhagic strokes. When atherosclerosis affects the renal arteries, it may impair kidney function and cause systemic hypertension. Additionally, atherosclerosis is associated with peripheral arterial occlusive disease and severe limb ischemia (Wu et al. 2017).

Natural medicines including herbals have been used to treat stroke and other cardio‐cerebrovascular diseases from the beginning of human civilization in Mesopotamia (Scurlock 2021). In recent years, traditional Chinese medicine (TCM) has been adopted in the treatment of various cardiovascular diseases in the context of modern clinical practice, with an increasing research interest in the underlying pharmacological mechanisms. Many ancient TCM prescriptions are studied and refined based on modern chemistry findings, where different components are combined to enhance the treatment efficacy and reduce the side effects. Many natural medicines have shown treatment efficiency for atherosclerosis‐related diseases and been accepted as complementary and alternative therapies of some cardiovascular diseases in many countries (Sadiq 2023).

TCM typically functions as complex mixtures of herbs, where the therapeutic benefits arise from synergistic interactions among multiple bioactive components rather than isolated compounds. The effects of individual constituents (e.g., flavonoids, saponins) on specific pathways may not fully capture the holistic effects of TCM formulations. Compared with single‐target chemical drugs (e.g., statins for lowing lipid profiles), the multi‐target, multi‐pathway approach of TCM aligns with the complex pathogenesis of atherosclerosis, which involves oxidative stress, inflammation, and lipid dysregulation. TCM's holistic modulation of different pathways offers unique therapeutic potential but also presents challenges in mechanistic elucidation and standardization. Atherogenesis is driven by intertwined pathological processes—oxidative stress begets inflammation, which in turn exacerbates oxidative damage—making it challenging to delineate whether TCM's modulation of a particular pathway is a primary or secondary effect. Different mechanisms work as a dynamic network rather than linear causality. In addition, the safe use of TCM necessitates expert guidance to mitigate risks of overdose and herb‐drug interactions. There is a high need for a comprehensive review of TCM‐based natural medicines and their extracts in the treatment of atherosclerosis, with in‐depth analysis of the pharmacological mechanisms.

To address this research gap, this review systematically catalogs TCM extracts, compounds, and herbal medicines for atherosclerosis. The latest findings are contextualized within the evolving landscape of atherosclerosis research. This review not only summarizes existing evidence but also critically evaluates the translational gaps between preclinical findings and clinical applications, offering a roadmap for future research.

## 2 Pathology of Atherosclerosis: The Role of TCM

### 2.1 Pathology of Atherosclerosis

The classical theory of atherosclerosis development divides the disease into three stages: onset, progression, and acute exacerbation. The key pathological features of atherosclerosis include lipid accumulation, local inflammation, smooth muscle cell proliferation, apoptosis, necrosis, and fibrosis. Atherosclerosis begins with endothelial dysfunction caused by injury to endothelial cells (ECs), which triggers the filtration of low‐density lipoprotein (LDL) into the endothelium and the recruitment of blood monocytes. These monocytes enter the damaged endothelium, where they differentiate into macrophages. Both macrophages and smooth muscle cells migrate into the endothelium, engulf lipids, and transform into foam cells. The accumulation and apoptosis of foam cells lead to the formation of atherosclerotic plaques, resulting in arterial lumen narrowing and occlusion (Wang, Wang, et al. 2024; Zhang, Qiu, et al. 2022).

Lipid‐lowering drugs, such as statins including simvastatin, atorvastatin (Lipitor), and cotrimoxazole, can reduce blood lipid levels by competitively inhibiting HMG‐CoA reductase (the rate‐limiting enzyme in cholesterol synthesis). Statins reduce intracellular cholesterol synthesis and increase the clearance of serum cholesterol by stimulating LDL receptors on cell surfaces. Clinically, these drugs are primarily used to lower cholesterol, particularly LDL‐cholesterol (LDL‐C), and slow the progression of atherosclerosis. However, lipid‐lowering drugs alone are insufficient to fully prevent or reverse the condition (Mu et al. 2025).

**FIGURE 1:**
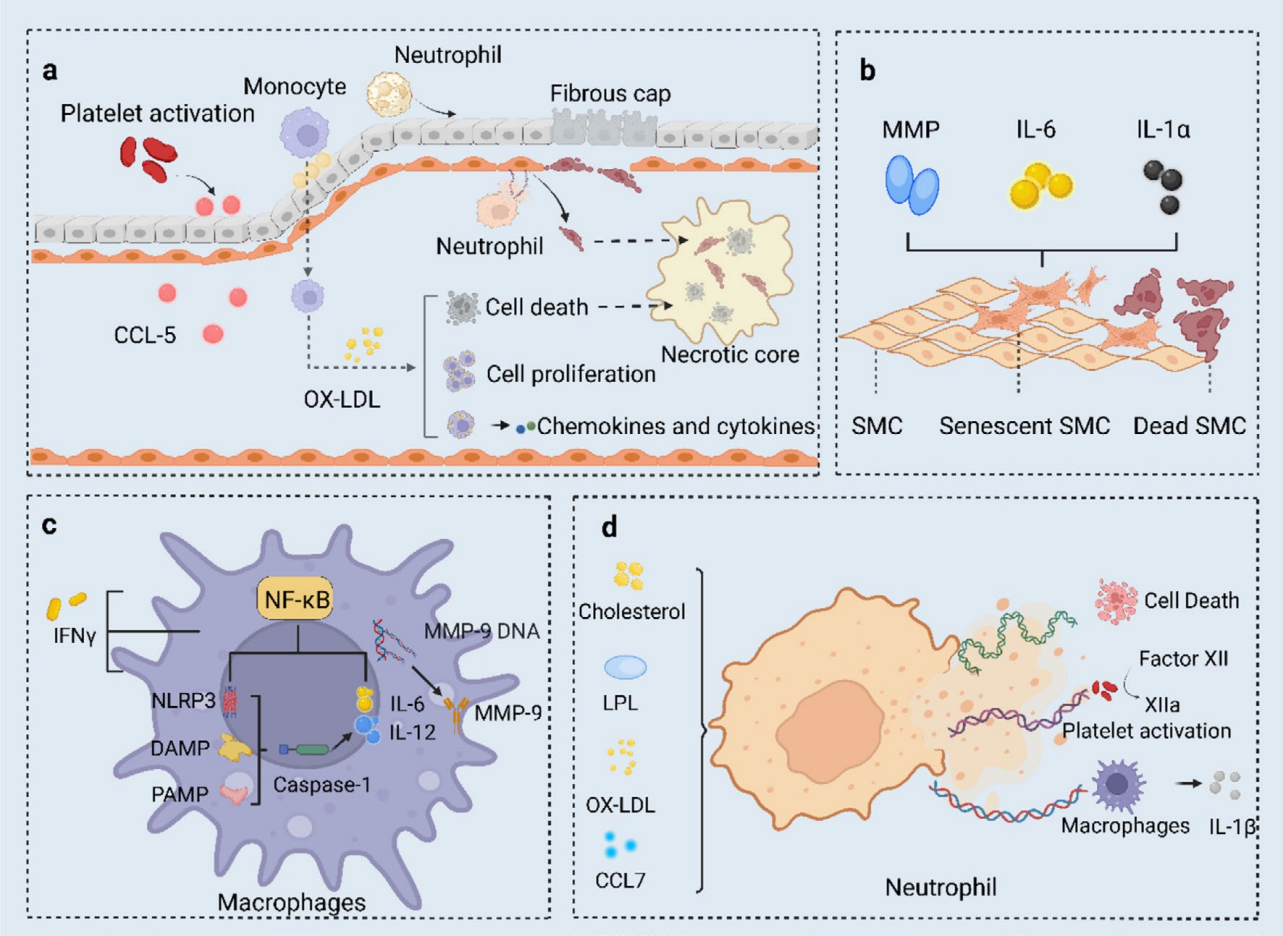
Inflammatory processes in atherogenesis. (a) Activated platelets release the chemokine CCL5, which promotes the adhesion of monocytes and neutrophils, facilitating their infiltration into the vessel wall. (b) Cholesterol accumulation leads to SMC death, and the loss of SMCs decreases the production of extracellular matrix, further exacerbating SMC apoptosis. (c) The priming and activation of the NACHT, LRR, and PYD domains‐containing protein 3 (NLRP3) inflammasomes occur through several mechanisms. Priming is initiated by cholesterol crystals and interferons such as IFNγ, which activate the NF‐κB signaling pathway. This, in turn, upregulates the transcription of NLRP3 and pro‐IL‐1β. (d) The release of neutrophil extracellular traps (NETs) is stimulated by cholesterol crystals, lipopolysaccharides (LPS), modified lipids, and chemokines such as CCL7. NETs exhibit cytotoxic effects through NET‐bound histones, which stimulate the NLRP3 inflammasome in macrophages.

The pathogenesis of atherosclerosis is highly complex, with widely recognized contributing factors including high blood lipid levels, elevated blood glucose, obesity, hypertension, smoking, excessive alcohol consumption, and genetic predisposition (Ugusman et al. 2021; Wolf and Ley 2019). Reactive oxygen species (ROS) play a central role in inflammation by interacting with key molecular pathways, such as nuclear factor kappa B (NF‐κB), endoplasmic reticulum (ER) stress, and mitochondrial dysfunction, all of which are involved in atherogenesis (Batty et al. 2022). Various cell types contribute to the inflammatory response in the intima‐media layer, including dendritic cells, T and B lymphocytes, vascular smooth muscle cells (VSMCs), ECs, and different subsets of macrophages (Blagov et al. 2023). Among these, macrophages regulate both inflammatory and metabolic signaling of atherogenesis (Engelen et al. 2022). As illustrated in Figure 1, in advanced atherosclerotic lesions, SMCs from the medial layer migrate to the forming fibrous cap. Excessive lipid accumulation within these SMCs leads to apoptosis. If apoptotic cells are not promptly cleared, they progress to necrosis. Concurrently, monocyte‐derived macrophages engulf modified lipids, triggering the secretion of inflammatory chemokines. Excess lipid uptake in macrophages drives their proliferation and can eventually lead to cell death. Senescent SMCs release pro‐inflammatory cytokines and matrix‐degrading enzymes such as matrix metalloproteinases (MMPs), which contribute to plaque instability.

### 2.2 Mechanisms of TCMs in Treating Atherosclerosis

**FIGURE 2:**
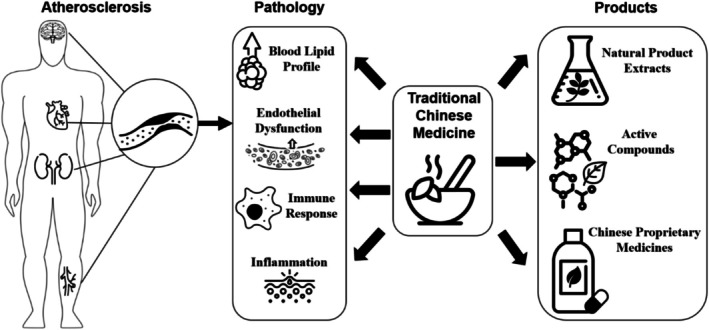
The pathology of atherosclerosis and role of TCM and its modern products in the treatment of atherosclerosis.

The main mechanisms of TCMs in reducing atherosclerosis include regulation of blood lipids, anti‐lipid peroxidation, anti‐polymerization, anti‐coagulation and pro‐fibrinolysis, inhibition of smooth muscle cell proliferation, and protection of vascular endothelial function. Besides traditional processing methods, modern technologies provide different TCM products including natural product extracts, active compounds, and proprietary Chinese medicines, which may condense the bioactive materials and improve the efficiency of TCM in treating atherosclerosis (Figure 2).

### 2.3 Gut Microbiota Modulation in TCM Treatment of Atherosclerosis

Gut microbiota has been recognized as a key susceptibility factor for atherosclerosis. The human gut microbiota is composed of six major phyla: Firmicutes, Bacteroidetes, Actinomycetes, Ascomycetes, Fusobacteria, and Verrucomicrobiales, with Firmicutes and Bacteroidetes representing the predominant bacterial populations. The gut microbiome also harbors a diverse fungal community, with Candida, *Saccharomyces cerevisiae* , Malassezia, and Cladosporium being the most frequently studied genera, as well as viruses, bacteriophages, and archaea, all of which contribute to host health and disease (Zhu et al. 2020).

In recent years, modulating the intestinal microbiota to inhibit the development of atherosclerosis is an emerging research hotspot in the field of natural product‐based therapeutics. Utilizing a high‐fat diet‐induced atherosclerosis model in ApoE^−/−^ mice, Dai et al. demonstrated that capsaicin supplementation significantly modulated the gut microbiota and fecal metabolite profile. Specifically, capsaicin increased the relative abundance of Turicibacter, Odoribacter, and Ileibacterium, while concurrently reducing levels of deoxycholic acid, cholic acid, hypoxanthine, and stercobilin in feces. These microbial and metabolic alterations were associated with a marked attenuation in the progression of atherosclerotic lesions, suggesting a potential anti‐atherogenic effect of capsaicin via gut microbiota modulation (Dai et al. 2022). Shi et al. demonstrated that berberine (BBR) attenuated high‐fat diet‐induced atherosclerosis through modulation of the gut microbiota. Notably, the trimethylamine TMA‐FMO3‐TMAO pathway was implicated as a potential mechanistic link, highlighting the role of gut microbial metabolism in mediating the anti‐atherogenic effects of BBR (Shi, Hu, et al. 2018).

A growing body of evidence indicates that various phytochemicals derived from TCM herbal sources exert anti‐atherosclerotic effects by modulating the composition and function of the intestinal microbiota. Ji et al. found that Neixiao‐ruanmai decoction No 2 attenuated the progression of coronary atherosclerosis, accompanied by significant modulation of the gut microbiota. Mechanistically, it suppressed the intestinal Toll‐like receptor 4 (TLR4)/NF‐κB signaling pathway and upregulated the expression of intestinal tight junction proteins, suggesting a multifaceted protective role involving both microbial and intestinal barrier regulatory mechanisms (Ji et al. 2025). Wang et al. found that Qing‐Xin‐Jie‐Yu Granule can reshape the gut microbiota and modulate the associated bile acid metabolomic profile, in part through the upregulation of hepatic bile acid synthesis enzymes, including CYP7A1 and CYP27A1. Concurrently, it downregulated the mRNA expression of ileal fibroblast growth factor 15 (FGF15) and *β*‐Klotho, thereby promoting de novo bile acid synthesis and enhancing cholesterol catabolism and excretion, which collectively contribute to its anti‐atherosclerotic efficacy (Wang, Guan, et al. 2022). These microbiota‐targeted interventions contribute to the attenuation of atherosclerotic progression, underscoring the therapeutic potential of TCM in gut‐mediated cardiovascular protection.

## 3 Anti‐Atherogenic Herbal Medicines

Many anti‐atherogenic herbal medicines have been identified from TMC literature based on the recorded functions of “romoting blood circulation for removing blood stasis.” Some of them have been validated and applied in modern clinical practice (Table 1).

**TABLE 1:** Summary of anti‐atherogenic herbal medicines.

Plant	Model	Mechanism of action	References
*Prunella vulgaris* L.	High glucose (HG)‐induced expression of cell adhesion molecules in human umbilical vein endothelial cells (HUVEC)	Anti‐inflammatory and antioxidant (Inhibition of HL‐60 cell adhesion to endothelial cells; reduction of ROS levels; suppression of NF‐κB p65 activation; Induction of Akt phosphorylation; activation of HO‐1, eNOS and Nrf2 pathways).	Hwang et al. (2012)
*Ginkgo biloba* L.	New Zealand white rabbits and arsenite‐induced SD rats	Anti‐inflammatory and antioxidant (reduction of intracellular inflammatory factors and ROS; regulation of blood lipid levels).	Chen et al. (2024); Zhang, Chen, Sun, et al. 2024
*Panax notoginseng* (Burk.) F. H. Chen	ox‐LDL‐induced HAECs and High‐fat chow‐induced SD rats	Anti‐inflammatory effects (through regulation of IL‐1*β*, IL‐1, MMP‐2 and MMP‐9 expression).	Xiao et al. (2024); Xie et al. (2024)
*Erigeron breviscapus* (Vant.) Hand.‐Mazz	Acetic acid‐induced peripheral pain mice and MIA‐Induced Osteoarthritis rats.	Anti‐inflammatory (through regulation of IL‐1*β*, IL‐6, NOS2 and MMP‐13).	Jo et al. (2024)
*Panax ginseng* C. A. Mey	BALB/c mice	Antioxidant and anti‐apoptotic (through regulation of SOD and GSH activities, Bcl‐2 upregulation, and Bax downregulation).	Shin et al. (2002)
*Astragalus membranaceus* (Fisch.) Bunge	Nrf2 activators‐induced the expression of CD36 gene in RAW264.7 mouse macrophages	Anti‐apoptotic and antioxidant (through reduction of ROS production, down‐regulation of Bax/Caspase‐9/Caspase‐3, and up‐regulation of HO‐1/Nrf2).	Maruyama et al. (2008)
*Ganoderma tsugae* Murr.	Isoprenaline‐induced H9c2 cells and H_2_O_2_‐induced H9c2 cells.	Anti‐apoptotic and antioxidant (through suppression of ROS levels and blockade of p38 activation).	Kuok et al. (2013)
*Arctium lappa* L.	High‐fat chow‐induced SD rats	Anti‐atherogenic (through activation of the AMPK/ACC/CPT‐1 pathway).	Ruan et al. (2022)
*Carthamus tinctorius* L.	Hypoxia and glucose deprivation‐induced H9c2 cells.	Provides cardiomyocyte protection (through STAT3‐mediated upregulation of VEGFA and HIF‐1α transcription).	Zhong et al. (2024)
*Morus alba* L.	Diabetic rat model.	Hypolipidemic and hypoglycemic (through regulation of blood glucose and lipid levels).	Li et al. (2015)

### 3.1 *Panax notoginseng* (Burk.) F. H. Chen

*Panax notoginseng* (Burk.) F. H. Chen, a traditional Chinese herb known for its properties in promoting blood circulation, removing blood stasis, and unblocking meridians, has long been used in the treatment of cardiovascular and cerebrovascular disease sequelae. Research has shown that *Panax notoginseng* saponins (PNS) can inhibit or slow the progression of atherosclerosis through their anti‐inflammatory, antiplatelet, and antioxidant effects. PNS exert their anti‐atherogenic effects by reducing the expression of IL‐1*β*, IL‐1, integrins, matrix metalloproteinase‐2 (MMP‐2), and MMP‐9 (Liu et al. 2022). PNS are classified into two main types: 20(S)‐protopanaxadiol‐type saponins (PDS) and 20(S)‐protopanaxatriol saponins (PTS), based on their saponin structure (Chu and Zhang 2009). Key PDS include ginsenosides Ra1, Rb1, Rb2, Rc, and Rd, while prominent PTS include ginsenosides Re, Rf, Rg1, and Rg2 (Chu and Zhang 2009). Studies have shown that PDS plays a dominant role in the anti‐atherogenic process, whereas PTS show no significant inhibitory effect on atherogenesis. Furthermore, PDS and PTS exhibit distinct or even opposing biological activities: PDS possess hemolytic properties, while PTS are anti‐hemolytic. For instance, Re and Rg1 promote angiogenesis, whereas Rb1, Rg3, and Rh2 inhibit it. Notably, PDS have been found to inhibit NF‐κB‐mediated vascular inflammation and protect against TNF‐*α*‐induced inflammation in human umbilical vein endothelial cells (HUVECs) more effectively than PTS, indicating that PDS are the key active constituents responsible for the anti‐atherogenic effects of PNS (Wang et al. 2011).

### 3.2 *Panax ginseng* C. A. Mey

Research has shown that ginsenoside Rg3, extracted from ginseng, enhances lipocalin secretion by regulating the lipocalin pathway. This action helps prevent the accumulation of cardiovascular lipids and mitigates mitochondrial dysfunction in cardiac cells, reducing the risk of cardiovascular sclerosis (Zhang, Yu, et al. 2023). Research has shown that ginsenoside Rg3, extracted from ginseng, enhances lipocalin secretion by regulating the lipocalin pathway. This action helps prevent the accumulation of cardiovascular lipids and mitigates mitochondrial dysfunction in cardiac cells, reducing the risk of cardiovascular sclerosis. Additionally, animal studies have demonstrated that ginseng polysaccharides can modulate the activity of superoxide dismutase (SOD) enzymes and glutathione (GSH), significantly decrease the expression of B‐cell lymphoma‐2 (Bcl‐2) and Bax proteins in rats, and improve blood lipid profiles, thereby inhibiting the progression of atherosclerosis (Guo et al. 2021).

### 3.3 *Arctium lappa* L.

The root of *Arctium lappa* L., commonly consumed as food or tea in various countries, has been shown to improve lipid metabolism in response to high‐fat and high‐glucose diets. The ethanolic extract of *Arctium lappa* L. enhances hepatic fatty acid *β*‐oxidation and alleviates hepatic steatosis through the activation of the AMPK/ACC/CPT‐1 pathway, suggesting potential anti‐atherogenic properties (Ma et al. 2022). Additionally, *Arctium lappa* L. polysaccharides can inhibit clot formation by reducing oxidative stress and regulating platelet activity, thereby preventing atherosclerosis (Ruan et al. 2022).

### 3.4 *Ginkgo biloba* L.

*Ginkgo biloba* L. is traditionally known for its properties of promoting blood circulation, reducing fat, and resolving turbidity. Animal studies have demonstrated that *Ginkgo biloba* extract possesses anti‐inflammatory, antioxidant, and antitumor activities (Pu et al. 2024; Yu et al. 2023). Ginkgolides isolated from *Ginkgo biloba* exhibit strong anticoagulant and antithrombotic effects (Zhang, Chen, Sun, et al. 2024). Furthermore, *Ginkgo biloba* extract can protect against hepatocyte damage and atherosclerosis by reducing oxidative stress and inflammation (Chen et al. 2024).

### 3.5 *Astragalus membranaceus* (Fisch.) Bunge

Astragalus, a TCM known for its ability to tonify qi and nourish blood, has demonstrated promising therapeutic effects on atherosclerosis, particularly through its total flavonoids. The key bioactive components of Astragalus include Astragalus polysaccharide (APS), astragaloside IV (ASIV), and Astragalus extract (ARE). APS, in particular, exhibits antioxidant, antibacterial, and antitumor properties and functions as an immune modulator (Li et al. 2010; Xu et al. 2023). APS reduces the production of ROS, downregulates the expression of Bax, Caspase‐9, and Caspase‐3, while upregulating heme oxygenase 1 (HO‐1) and nuclear factor erythroid 2‐related factor 2 (Nrf2), thereby inhibiting apoptosis and protecting ECs from oxidative damage. APS also enhances the expression of the transcriptional regulator KLF2, which activates Nrf2 in ECs, providing cardiovascular protection (Maruyama et al. 2008; Yang et al. 2016).

### 3.6 *Erigeron breviscapus* (Vant.) Hand.‐Mazz

*Erigeron breviscapus* attenuates inflammatory responses by inhibiting key inflammatory cytokines, such as IL‐1*β*, IL‐6, nitric oxide synthase 2 (NOS2) and MMP‐13. These anti‐inflammatory effects suggest that *Erigeron breviscapus* may help slow the progression of atherosclerosis by reducing inflammation (Jo et al. 2024).

### 3.7 *Carthamus tinctorius* L.

*Carthamus tinctorius* L., commonly used in TCM for treating cardiovascular diseases, is known for its ability to activate blood circulation and remove blood stasis. In addition, it is used to alleviate depression, calm the mind, cool the blood, and detoxify. Clinically, it is applied in the treatment of post‐partum blood stasis, depression, and certain mental disorders (Zhou et al. 2014). Studies have demonstrated that *Carthamus tinctorius* seeds can inhibit the progression of atherosclerosis by reducing oxidative stress. Network pharmacology analysis has revealed that the cardiovascular protective effects of *Carthamus tinctorius* are closely linked to the platelet activation pathway. Six key molecular targets associated with these effects have been identified, including PRKACA, PIK3R1, MAPK1, PPP1CC, PIK3CA, and SYK (Yu et al. 2019).

### 3.8 *Ganoderma tsugae* Murr

A peptidoglycan‐like compound found in *Ganoderma tsugae* Murr. extract (GTE) has been shown to promote the translocation of Nrf2 to the nucleus, inducing the expression of HO‐1 and thioredoxin reductase‐1 (TrxR‐1) in bovine arterial endothelial cells (BAEC). Furthermore, long‐term treatment with GTE upregulates GSH, SOD‐1, and HO‐1, while reducing vascular permeability and mitigating the genotoxicity and apoptosis of ECs caused by particulate matter (PM2.5). This protective effect on the vascular endothelium helps prevent the progression of atherosclerosis (Wei et al. 2009).

### 3.9 *Morus alba* L.

As recorded in the Compendium of Materia Medica (Bencao gangmu, 1596), *Morus alba* L. is traditionally known for its detoxifying, anti‐inflammatory, and blood‐nourishing effects, and is used to treat conditions such as liver and kidney deficiency, dizziness, tinnitus, and insomnia. *Morus alba* polysaccharides possess antioxidant, anti‐inflammatory, antibacterial, and blood pressure‐lowering properties (Wang and Huang 2024). Rich in anthocyanins, *Morus alba* significantly enhances the activity of VECs, thereby reducing the incidence of atherogenesis. Additionally, *Morus alba* leaves exhibit antioxidant, hypoglycemic, hepatoprotective, and immunomodulatory effects (Hu et al. 2024).

### 3.10 *Prunella vulgaris* L.

*Prunella vulgaris* L., a well‐known traditional Chinese herb, is recognized for its ability to clear heat, reduce swelling, and disperse nodules. Research has shown that the aqueous extract of *Prunella vulgaris* (APV) inhibits the adhesion of HL‐60 cells to arterial ECs, reduces ROS levels, and downregulates the expression of ICAM‐1, VCAM‐1, and endothelial‐leukocyte adhesion molecule‐1 (ELAM‐1). Additionally, APV inhibits the activation of NF‐κB p65, induces Akt phosphorylation, and activates HO‐1, endothelial nitric oxide synthase (eNOS), and Nrf2, providing protective effects against vascular inflammation and oxidative stress (Hwang et al. 2012).

In summary, traditional Chinese medicinal herbs are characterized by their diverse composition, which confers multi‐target and multi‐mechanism properties. Current research predominantly focuses on the bioactivity of herbal extracts. Future studies should prioritize the investigation of the individual components of Chinese medicinal herbs, combined with approaches such as transcriptomics and metabolomics, to elucidate their specific mechanisms of action.

## 4 Anti‐Atherogenic Natural Product Extracts and Bioactive Components

**FIGURE 3:**
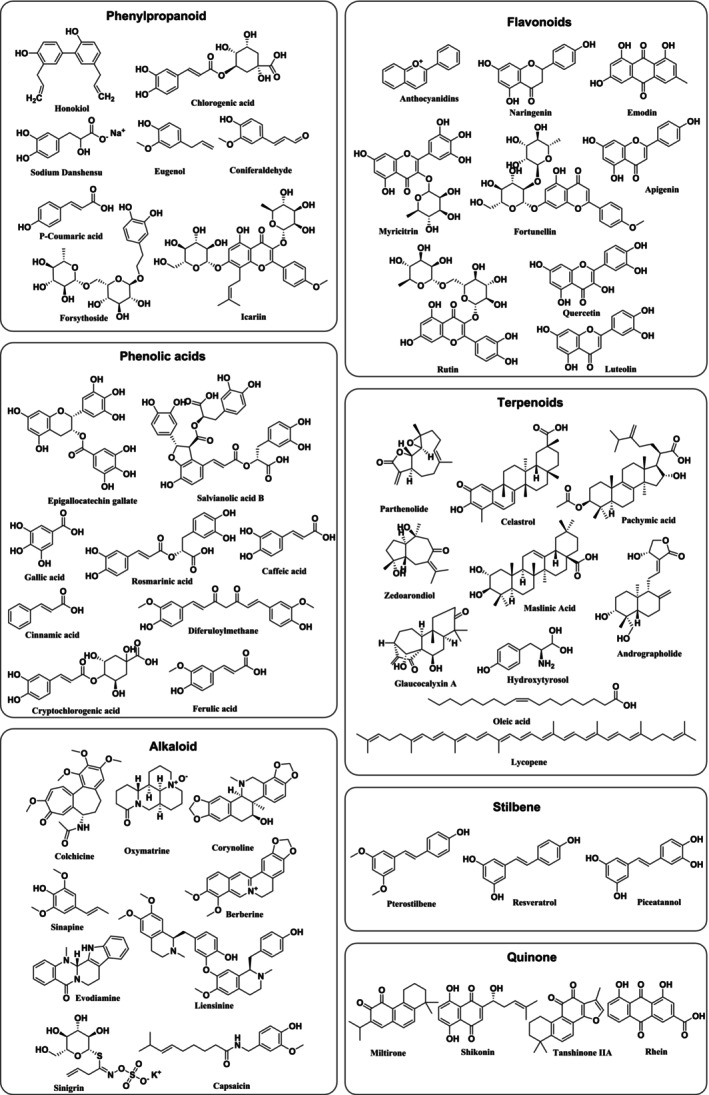
Different types of bioactive components extracted from TCM for treating atherosclerosis.

Some bioactive components in natural products such as phenylpropanoids, flavonoids, phenolic acids, terpenoids, alkaloids, stilbene, and quinone have therapeutic effects on atherosclerosis with different mechanisms (Table 2, Figure 3).

**TABLE 2:** Summary of natural products for cardiovascular disease treatment.

Compound category	Compounds	Model	Mechanism of action	References
Phenylpropanoid	Honokiol, Chlorogenic acid, Sodium Danshensu, Eugenol, Coniferaldehyde, Forsythoside, P‐Coumaric acid, Icariin	Cecal ligation and puncture (CLP) or LPS‐induced mice. LPS‐induced HL‐1 and AC16 cells. Palmitic acid‐induced HepG2 cells.	Anti‐inflammatory and antioxidant (through inhibition of IL‐6/TNF‐α production and ROS scavenging). Anti‐inflammatory and antioxidant (via Nrf2/HO‐1 pathway activation). Antioxidant (through LKB1/AMPK signaling activation).	Zhou et al. (2023); Miao and Xiang (2020); Zhang, Yang, Zhang, et al. (2024); Kiokias et al. (2020)
Flavonoids	Anthocyanidins, Naringenin, Myricitrin, Fortunelli, Emodin, Quercetin, Luteolin, Apigenin, Rutin	ox‐LDL injured human umbilical vein endothelial cells. H_2_O_2_‐induced human umbilical vein endothelial cells. Atherosclerotic ApoE^−/−^ mice model.	Anti‐inflammatory and antioxidant (via Nrf2/HO‐1 pathway activation). Anti‐inflammatory (through inhibition of IL‐6/TNF‐α production). Anti‐inflammatory (through TLR4‐NF‐κB‐NLRP3 axis regulation).	Luo et al. (2017); Weng et al. (2023); Liu et al. (2024); Sthijns et al. (2017)
Phenolic acids	Phenolic acids, Epigallocatechin gallate, Curcumin, Gallic acid, Rosmarinic acid, Caffeic acid, Salvianolic acid B, Ferulic acid, Cinnamic acid, Cryptochlorogenic acid	H_2_O_2_‐induced human umbilical vein endothelial cells. ox‐LDL injured human umbilical vein endothelial cells. High‐fat chow‐induced SD rats.	Anti‐inflammatory (via inhibition of NF‐κB signaling and downstream cytokines TNF‐α/IL‐6/IL‐1*β*/IL‐8). Anti‐inflammatory and antioxidant (via Nrf2/HO‐1 pathway activation). Antioxidant (through ROS scavenging).	Xia et al. (2014); Lin and Zhang (2020); Zhao et al. (2020)
Terpenoids	Pachymic acid, Celastrol, Oleic acid, Maslinic acid, Andrographolide, Glaucocalyxin A, Zedoarondiol, Lycopene, Parthenolide, Hydroxytyrosol	LPS‐induced C57BL/6 mice. ox‐LDL‐induced human umbilical vein endothelial cells.	Anti‐inflammatory and antioxidant (via Nrf2/HO‐1 pathway activation). Anti‐apoptotic (via inhibition of Bcl‐2/Bax/Caspase‐3 signaling).	Zhou et al. (2009); Lee et al. (2020); Sung et al. (2015); Marrero et al. (2023)
Alkaloid	Colchicine, Oxymatrine, Corynoline, Berberine, Liensinine, Sinapine, Sinigrin, Capsaicin, Evodiamine	Atherosclerotic ApoE^−/−^mice model. LPS‐induced human umbilical vein endothelial cells. Dextran sulfate sodium‐induced mice.	Anti‐apoptotic (through MMPs downregulation and Bcl‐2/Bax ratio modulation). Anti‐inflammatory (through enhanced cholesterol metabolism and lipid balance regulation). Anti‐inflammatory and antioxidant (via Nrf2/HO‐1 pathway activation).	Nidorf et al. (2013); Liu et al. (2018); Zhang, Lang, et al. (2023); Li et al. (2009)
Stilbene	Pterostilbene, Resveratrol, Piceatannol	Oil acid and palmitic acid‐induced HepG2 cells. Palmitic acid‐induced human umbilical vein endothelial cells. PMA, LPS, IL‐8‐induced human neutrophils.	Anti‐inflammatory and antioxidant (via Nrf2/HO‐1 pathway activation). Anti‐inflammatory (through downregulation of IL‐6 and TNF‐α). Antioxidant (via AMPK‐mTOR pathway‐mediated ROS inhibition).	Stewart et al. (2010); Shen et al. (2023); Pan et al. (2018)
Quinone	Miltirone, Tanshinone IIA, Rhein, Shikonin	High‐fat diet‐fed C57BL/6J mice. LPS‐induced C57BL/6J mice. LPS‐induced RAW264.7 cells. ox‐LDL‐induced EA. hy926 cells.	Anti‐inflammatory and antioxidant (via Nrf2/HO‐1 pathway activation). Anti‐inflammatory and antioxidant (via HDL‐C elevation and LDL‐C reduction). Anti‐inflammatory (via downregulation of IL‐1*β*/NLRP3 and modulation of IL‐10).	Zhang, Zhang, et al. (2016); Gong et al. (2009); Zhong et al. (2021)

**FIGURE 4:**
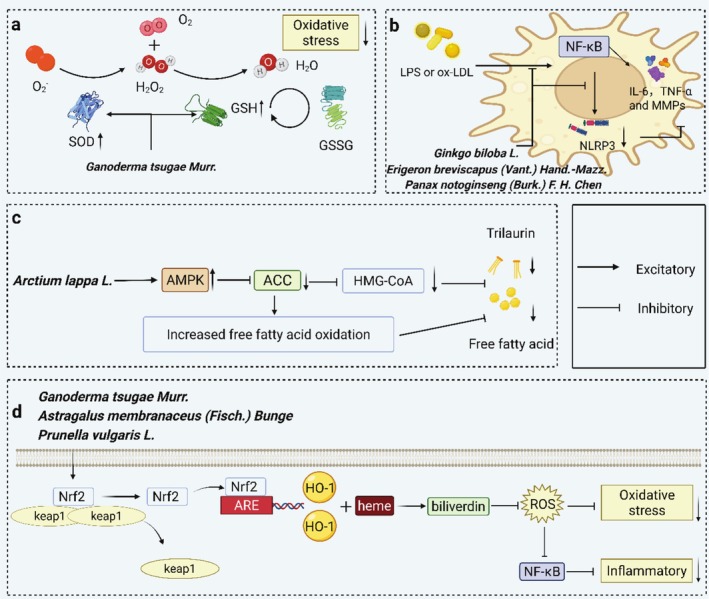
Mechanisms of TCMs in reducing inflammatory response during atherogenesis. (a) Inhibits oxidative stress by increasing SOD activity as well as increasing GSH content. (b) By inhibiting NF‐κB and NLRP3, it reduces the expression of inflammatory factors and thus suppresses inflammation. (c) Regulates lipid fatty acid and cholesterol metabolism through the AMPK/ACC signaling pathway. (d) Inhibits inflammation and oxidative stress through the Nrf2/HO‐1 signaling pathway.

The mechanism of most compounds against atherosclerosis is that scavenging free radicals inhibits the production of inflammatory factors through activation of the Nrf2/HO‐1 signaling pathway, which in turn inhibits the inflammatory response and oxidative stress (Figure 4) (Saha et al. 2020).

### 4.1 Phenylpropanoids

**FIGURE 5:**
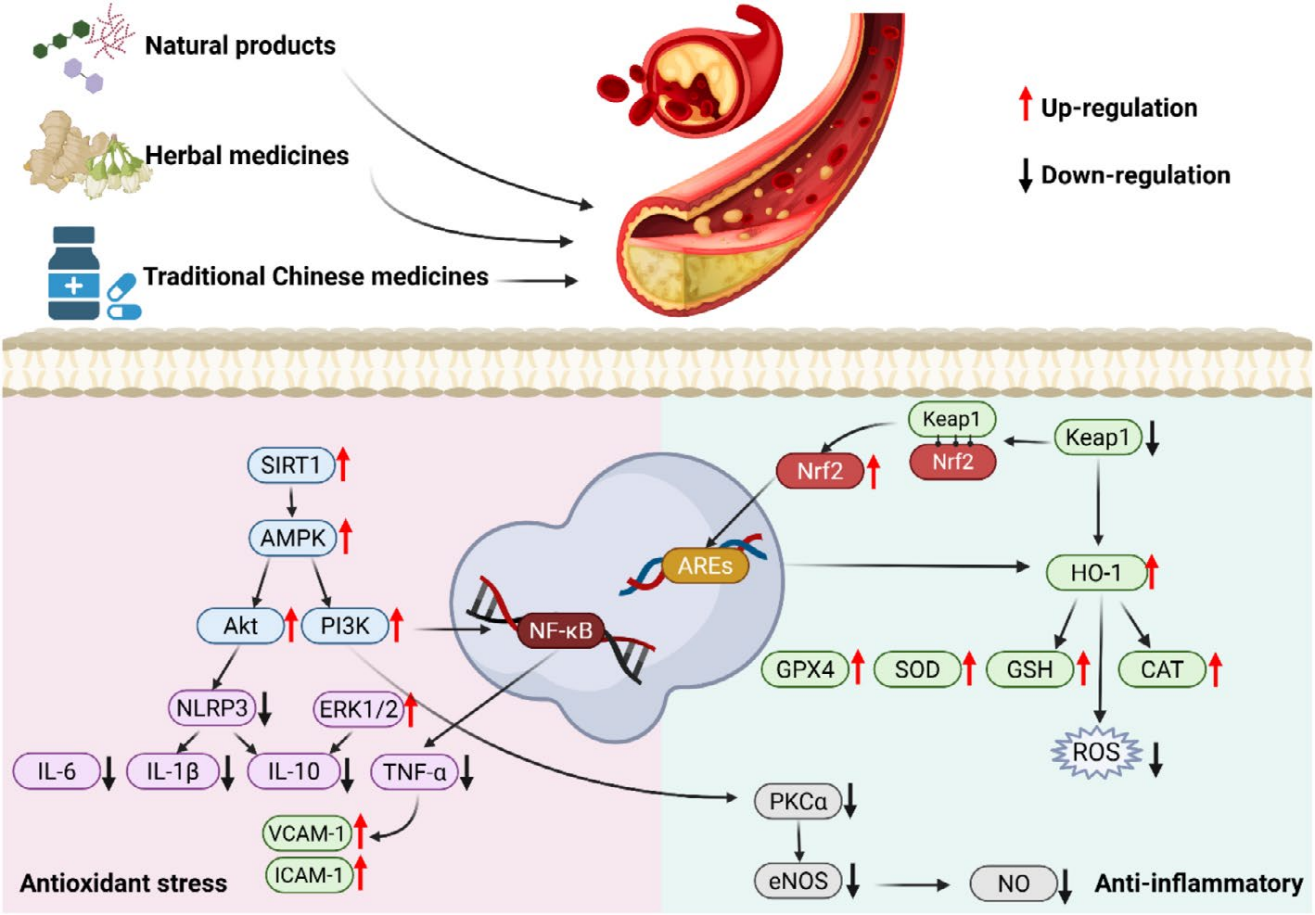
Mechanisms of natural product and TCM on Atherosclerosis. The natural product demonstrates therapeutic potential against atherosclerosis by concurrently modulating redox homeostasis and immune responses. (1) inhibiting NLRP3 inflammasome activation in macrophages, reducing IL‐1β secretion and lipoatrophy; (2) downregulating VCAM‐1/ICAM‐1 in HUVECs, attenuating leukocyte adhesion; and (3) modulating TLR4‐NF‐κB/MAPK pathways to suppress pro‐inflammatory cytokines (TNF‐α, IL‐1β). Additionally, stabilizes plaques via PI3K/AKT‐mediated caspase‐3 and NF‐κB regulation.

Phenylpropanoid compounds are widely distributed across various plant species and represent key active constituents in many medicinal plants. Certain phenylpropanoids exhibit antioxidant, anti‐inflammatory, and other biological activities. Among the various phenolic compounds discussed in this study, chlorogenic acid, danshensu sodium, and forsythoside exhibit antioxidant activity through the Nrf2/HO‐1 signaling pathway (Li, Li, et al. 2024; Miao and Xiang 2020; Zhang, Yang, Zhang, et al. 2024) (Figure 5), thereby contributing to their anti‐atherosclerotic effects. The other compounds and their respective mechanisms of action will be further elaborated in the subsequent sections.

Honokiol is a bioactive phenylpropanoid with antioxidant, anti‐inflammatory, anti‐angiogenic, and anticancer properties (Liu, Xun, et al. 2023; Luo et al. 2024). Animal experiments have shown that honokiol can protect VECs by reducing oxidative stress in rats, thereby preventing the progression of atherosclerosis (Zhou et al. 2023).

Chlorogenic acid, another phenylpropanoid, is produced during aerobic respiration and is commonly found in higher dicotyledonous plants and ferns. It demonstrates antioxidant, antimicrobial, antitumor, and anti‐inflammatory activities (Xiong et al. 2023). With five active hydroxyl groups and one carboxyl group, chlorogenic acid readily reacts with free radicals, inhibiting their formation and exhibiting strong antioxidant activity. Chlorogenic acid has been demonstrated to effectively reduce oxidative stress in animal models, thereby alleviating the progression of atherosclerosis (Miao and Xiang 2020).

Danshensu sodium exhibits anti‐inflammatory, antioxidant, antibacterial, and cardiovascular effects. It has been demonstrated that danshensu sodium can reduce oxidative stress, suppress inflammation, and protect VECs from atherosclerotic plaque formation (Zhang, Yang, Zhang, et al. 2024). Additionally, danshensu sodium reduces ROS production, thereby enhancing antioxidant defenses and preventing apoptosis in VECs.

Eugenol, a phenylpropanoid extracted from *Syzygium aromaticum* (L.) Merr. & L.M. Perry, inhibits ROS production induced by phorbol myristate acetate (PMA) or *N*‐formyl‐methionyl‐leucyl‐phenylalanine (fMLF) in human neutrophils. Eugenol also inhibits LPS‐induced activation of MMP‐2 and MMP‐9, thereby reducing oxidative stress and protecting cardiovascular cells from oxidative damage (Chniguir et al. 2024).

Coniferaldehyde, extracted from pine resin, exhibits antioxidant, anti‐inflammatory, and lipid metabolism regulatory properties. Studies show that coniferaldehyde improves glucose and lipid metabolism in palmitic acid (PA)‐induced HepG2 cells via the LKB1/AMPK signaling pathway, regulating intracellular triglyceride and cholesterol levels (Gai et al. 2020). This suggests that coniferaldehyde may improve lipid metabolism in humans, thus reducing VEC damage and preventing or mitigating atherosclerosis.

Forsythoside, an active compound from *Forsythia suspensa* (Thunb.) Vahl, has anti‐inflammatory and blood circulation‐promoting activities. Early studies indicate that forsythoside reduces oxidative stress, thereby inhibiting apoptosis in the cardiovascular system. In addition, forsythoside modulates the HMGB1/TLR4/NF‐κB pathways to reduce inflammatory responses in rats (Li, Li, et al. 2024).

P‐Coumaric acid, a phenylpropanoid found in vegetables, cereals, fungi, and certain herbal medicines, exhibits antitumor, antibacterial, and antioxidant properties. Animal experiments have shown that p‐coumaric acid protects VECs by upregulating GSH expression, thereby reducing oxidative stress (Kiokias et al. 2020).

Icariin, the primary active component of *Epimedium brevicornu* Maxim., inhibits oxidative stress induced by ox‐LDL in HUVECs. Research suggests that icariin upregulates the expression of lncRNA H19, which suppresses apoptosis and migration of HUVECs, thereby slowing the progression of atherosclerosis (Liu, Xu, et al. 2021).

### 4.2 Flavonoids

Flavonoids are widely distributed in natural plants and herbs and have been applied in the treatment of various conditions, including cancer, cardiovascular diseases, and age‐related ailments. Humans commonly obtain flavonoids through their diet, where they serve as essential supplements, and these compounds are also used in the prevention and treatment of atherosclerosis‐related diseases. Anthocyanidins, naringenin, dihydromyricetin, and emodin have been reported to exert antioxidant effects by modulating the expression of proteins associated with the Nrf2/HO‐1 signaling pathway (Feng et al. 2019; Luo et al. 2017). Other compounds and signaling pathways involved will be discussed in subsequent sections.

Anthocyanidins, a representative class of flavonoids in plants, enhance VEC function, reduce the secretion of ROS and inflammatory factors, and increase the expression of HO‐1 and Nrf2 proteins (Li et al. 2021; Syeda et al. 2019). Studies have shown that ECs treated with anthocyanin‐mediated serum (AMS) are more resistant to hyperoxic stress.

Naringenin, another flavonoid, has potent vasoprotective effects, inhibiting EC apoptosis induced by high glucose or free fatty acids through the induction of HO‐1 expression and PI3K expression (Feng et al. 2019).

Dihydromyricetin (DMY), a flavonoid isolated from the vine plant genus *Ampelopsis*, exhibits antioxidant, antibacterial, anti‐inflammatory, and antitumor activities (Li et al. 2017). DMY has been found to activate Akt and ERK 1/2, restore membrane potential, and inhibit caspase‐3 activity and EC apoptosis, thereby protecting ECs from oxidative injury induced by ox‐LDL (Luo et al. 2017). Additionally, DMY mitigates cardiomyocyte hypertrophy by reducing angiotensin II‐induced ROS production, further acting as an antioxidant and protecting against atherosclerotic plaque formation (Meng et al. 2015; Song et al. 2017).

Myricitrin, a flavonol glycoside, has been shown to protect against oxidative stress‐induced apoptosis in VECs by inhibiting ERK and the phosphorylation of apoptotic proteins (Sun et al. 2013). It also exhibits anti‐inflammatory effects by significantly reducing the production of NO, TNF‐*α*, IL‐6, and MCP‐1 in macrophages, thereby inhibiting the progression of atherosclerosis (Qi et al. 2017; Xu et al. 2020).

Fortunellin, a flavonoid isolated from kumquat fruit, demonstrates anti‐inflammatory, hepatoprotective, and antioxidant properties (Shalaby et al. 2023; Weng et al. 2023). It has been shown to inhibit the TLR4/NF‐κB/NLRP3 pathway, a key receptor in the Toll‐like receptor family that mediates LPS‐stimulated tissue damage, apoptosis, and inflammation, thus protecting VECs and preventing thrombosis and atherosclerosis progression (Liu et al. 2024).

Emodin, a flavonol diglycoside, possesses antioxidant and antitumor properties (Shanmugam et al. 2017). It has been reported to protect ECs from H_2_O_2_‐induced oxidative stress (Sthijns et al. 2017).

Quercetin, a natural flavonoid found in the bark, flowers, leaves, seeds, and fruits of many plants, has demonstrated antioxidant, antibacterial, and antitumor effects. In mice, quercetin has been shown to slow atherosclerosis progression by modulating the apelin signaling pathway and upregulating the expression of apelin receptor proteins, PGC‐1*α*, and UCP1 (Liu, Zhang, et al. 2023).

Luteolin, found in various herbs, vegetables, and fruits, exhibits anti‐inflammatory, antioxidant, antiviral, antibacterial, and antitumor activities (Hakem et al. 2023; Liu and Li 2024; Shaukat et al. 2024). Animal studies have demonstrated that luteolin can inhibit platelet aggregation, reduce thrombus formation in mesenteric arteries, and lower oxidative stress, thereby protecting VECs and preventing atherosclerotic plaque formation (Ye et al. 2023).

Apigenin, a flavonoid prevalent in temperate‐zone plants such as celery, has anti‐tumor, antiviral, antibacterial, and cardiovascular protective effects (Li, Chen, et al. 2023; Li, Mao, et al. 2023; Sain et al. 2023) Animal studies have shown that apigenin effectively inhibits NLRP3 inflammasome activation in atherosclerotic plaques, reduces ROS production, and blocks p65 nuclear translocation in the NF‐κB pathway, thereby preventing further atherosclerosis progression (Weng et al. 2023).

Rutin, another widely distributed flavonoid, possesses anti‐inflammatory, hypoglycemic, antioxidant, renal, and hepatic protective properties (Zhang, Zheng, et al. 2023). Animal experiments have demonstrated that rutin decreases the expression of inflammatory markers such as IL‐6, IL‐8, and NF‐κB, thereby reducing inflammation and inhibiting the progression of atherosclerosis (Iqbal et al. 2024).

### 4.3 Phenolic Acids

Phenolic acids are widely distributed in higher plants as secondary metabolites and exhibit significant antioxidant and anti‐inflammatory properties. Numerous phenolic acids, such as epigallocatechin gallate and cryptochlorogenic acid, have been shown to alleviate intracellular oxidative stress by reducing the generation of ROS through upregulation of Nrf2 and HO‐1 expression (Shi, Zhang, et al. 2018; Zhao et al. 2020). Additionally, phenolic acids inhibit the expression of pro‐inflammatory factors such as NF‐κB, TNF‐*α*, IL‐6, IL‐1*β*, IL‐8, and the chemotactic protein MCP‐1. This inhibition further suppresses the activation of signaling pathways such as MAPK and NF‐κB, effectively dampening the inflammatory response.

Epigallocatechin gallate (EGCG), a powerful free radical scavenger derived from tea polyphenols, protects ECs from oxidative stress induced by H_2_O_2_ through a mechanism associated with increased HO‐1 expression. EGCG also exerts antioxidant and anti‐apoptotic effects against various stimuli, including PM2.5, H_2_O_2_, angiotensin II, and microcystin‐LR‐induced EC injury (Shi, Zhang, et al. 2018; Toniolo et al. 2015).

Curcumin, a natural phenolic acid antioxidant extracted from *Curcuma longa* and turmeric of the Zingiberaceae family, exhibits anti‐inflammatory, lipid‐lowering, and anti‐tumor properties (Gupta et al. 2013; Kah et al. 2023). It suppresses macrophage infiltration and the activation of the NF‐κB inflammatory pathway (Li et al. 2020). Curcumin also decreases the expression of pro‐inflammatory adipokines such as TNF, MCP‐1, and plasminogen activator inhibitor‐1 (PAI‐1), while inducing the expression of lipocalin, which can inhibit the progression of atherosclerosis (Xia et al. 2014).

Gallic acid, an organic phenolic acid found in plants such as *Rheum palmatum* , *Eucalyptus grandis* , and *Cornus officinalis* , has broad applications across fields such as food, biology, medicine, and chemical industries, due to its antibacterial, antioxidant, anti‐inflammatory, and anti‐tumor effects (Sun and Shahrajabian 2023). Animal studies have demonstrated that the anti‐inflammatory mechanism of gallic acid primarily involves the MAPK and NF‐κB pathways. By reducing the release of inflammatory cytokines, chemokines, adhesion molecules, and inhibiting cellular infiltration, gallic acid mitigates inflammation, protects VECs, and inhibits atherosclerotic plaque formation (Bai et al. 2021).

Rosmarinic acid, a water‐soluble natural phenolic compound isolated from rosemary ( *Rosmarinus officinalis* ) of the Lamiaceae family, possesses a wide range of biological activities, including antibacterial, antiviral, antioxidant, and antitumor effects (Ali et al. 2022; Guan et al. 2022; Ivanov et al. 2022). As a potent natural antioxidant, rosmarinic acid exhibits strong activity in protecting cells from free radical damage, thereby reducing the risk of cancer and atherosclerosis. Studies have shown that rosmarinic acid protects ECs by downregulating the p38/FOXO1/TXNIP pathway and inhibiting the activation of inflammasomes, thus preserving normal physiological functions and potentially preventing the progression of atherosclerosis (Nyandwi et al. 2020). In animal studies, rosmarinic acid has been found to preferentially reduce ROS activity in VECs, attenuate oxidative stress, and inhibit lipid accumulation and atherosclerotic plaque formation (Lin and Zhang 2020).

Caffeic acid, another natural phenolic acid, is known for its antibacterial and antioxidant properties and has diverse applications in the pharmaceutical and cosmetic industries. Caffeic acid suppresses ROS production and protects VECs. Research on HUVECs has demonstrated that caffeic acid effectively reduces the expression of IL‐8 and TLR4, inhibiting the NF‐κB signaling pathway, thereby protecting ECs and preventing the formation of atherosclerotic plaques (Mudau et al. 2012; Wang, Kaur, et al. 2022).

Salvianolic acid B, a phenolic compound formed by the condensation of three molecules of salvianolic acid with one molecule of caffeic acid, has significant pharmacological effects on various organs, including the heart, brain, liver, and kidneys. Animal studies have indicated that salvianolic acid B significantly attenuates LPS‐induced oxidative stress in rats by inhibiting the expression of pro‐inflammatory cytokines TNF‐*α* and IL‐6, thereby reducing the inflammatory response. Thus, it is hypothesized that salvianolic acid B possesses anti‐atherosclerotic properties (Guo et al. 2023).

Ferulic acid is a natural phenolic compound found in various medicinal plants, with particularly high concentrations in traditional Chinese herbs such as *Angelica sinensis*, Rhizoma Ligustici Chuanxiong, Ascophyllum officinale, and *Ziziphus jujuba* seeds. Clinical studies have demonstrated that ferulic acid can inhibit the production of ROS and improve lipid profiles, oxidative stress, and inflammation in patients with hyperlipidemia. Additionally, research has shown that ferulic acid can suppress inflammation induced by H_2_O_2_ and isoprenaline, reducing apoptosis in VECs. The study further indicated that ferulic acid protects cardiovascular cells from oxidative stress and inflammation‐induced injury by regulating the miR‐499‐5p/p21 signaling pathway (Sun et al. 2021).

Cinnamic acid, a class of phenolic compounds derived from cinnamon bark and benzoin, is widely used in the flavor, fragrance, food additive, and pharmaceutical industries. It possesses anticancer and antioxidant properties. Studies have shown that cinnamic acid can inhibit the expression of pro‐inflammatory cytokines such as TNF‐*α*, IL‐6, myeloperoxidase (MPO), and TLR‐4 in rats, thereby reducing inflammation and slowing EC apoptosis (Rezaei et al. 2024).

Cryptochlorogenic acid (CCA) is a natural phenolic compound known for its anti‐inflammatory, anti‐obesity, and anti‐cardiac hypertrophy effects. Research has demonstrated that CCA regulates the expression of pro‐inflammatory factors and promotes the nuclear translocation of Nrf2 in RAW 264.7 macrophages, significantly inhibiting NF‐κB activation. In summary, CCA can alleviate LPS‐induced inflammation by modulating the NF‐κB/MAPK signaling cascade and inhibit oxidative stress by facilitating Nrf2 nuclear translocation, thereby delaying the progression of atherosclerosis (Zhao et al. 2020).

### 4.4 Terpenoids

Terpenoids are a diverse class of natural compounds with significant therapeutic potential, widely distributed in various plants. Research has shown that pachymic acid (PA), oleic acid (OA), zedoarondiol, and lycopene exert their respective antioxidant activities primarily through the regulation of the Nrf2/HO‐1 signaling pathway (Liu, Yan, et al. 2021; Lu et al. 2014; Mao et al. 2018; Sung et al. 2015; Bergman et al. 2019). In addition, certain terpenoids exhibit anti‐inflammatory properties by inhibiting the NF‐κB pathway and regulating the expression of specific non‐coding RNAs.

PA, a triterpenoid isolated from *Poria cocos*, has been reported to inhibit ox‐LDL‐induced apoptosis and upregulate the expression of Caspase‐3, Bax, and Bcl‐2 in ECs (Zhou et al. 2009). Further studies have demonstrated that PA reduces oxidative stress by competitively inhibiting the activity of phospholipase A2 (PLA2), thereby mitigating PLA2‐mediated cellular damage (Liu, Yan, et al. 2021).

Celastrol, derived from *Ranunculus* species, activates Nrf2, inhibits Nox2 and AT1 receptor expression, upregulates ERK1/2 phosphorylation, and reduces ROS production in ECs, which helps attenuate angiotensin II‐induced endothelial injury (Li et al. 2017).

OA, a biologically active triterpenoid, exhibits strong antioxidant and anti‐inflammatory effects (Santa‐María et al. 2023). OA has been shown to reduce ROS production, upregulate HO‐1 expression, and enhance Nrf2 nuclear translocation in ECs, thus protecting them from oxidative damage. Since LOX‐1, a key receptor involved in ox‐LDL phagocytosis and degradation in VECs, plays a significant role in atherosclerosis development, its silencing has been shown to inhibit OA‐induced Nrf2 and HO‐1 expression, indicating that LOX‐1 is involved in OA's protective effects on ECs (Jiang et al. 2015).

Oleanolic acid has been reported to modulate the IL‐17/ERK/AKT signaling pathway, thereby ameliorating inflammatory responses in mice (Pan et al. 2025). In addition, Oleanolic acid can attenuate oxidative damage by suppressing ROS production and iron overload‐induced ferroptosis (Ouyang et al. 2023).

Maslinic acid, a natural compound found in olives, has been shown to induce Nrf2 nuclear translocation in LPS‐stimulated ECs, increasing Nrf2‐ARE binding and reducing IL‐1*α* production. At a concentration of 20 μM, maslinic acid inhibited COX‐2 and iNOS expression, decreased NF‐κB activity, and suppressed STAT‐1 phosphorylation. Moreover, RNA interference‐mediated inhibition of HO‐1 reversed maslinic acid‐induced reductions in iNOS/NO expression, highlighting its role in slowing or inhibiting the progression of atherosclerosis (Lee et al. 2020).

Andrographolide (ADH) inhibits TNF‐*α*‐induced oxidative stress by reducing ROS production, NADPH oxidase activation, Src phosphorylation, and ICAM‐1 expression, while increasing GSH levels and inducing the expression of HO‐1 and GCLM genes in EA.hy926 ECs (Lu et al. 2014). Another study highlighted ADH's protective effects against hypoxia‐induced damage in EA.hy926 cells (Lin et al. 2017). ADH also exerts anti‐inflammatory effects by inhibiting NF‐κB expression (Raghavan et al. 2012).

Glaucocalyxin A (Gau A), a diterpenoid from Isodon japonica, has been shown to protect ECs from H_2_O_2_‐induced damage. Studies revealed that Gau A downregulates endothelin‐1 (ET‐1) and iNOS mRNA expression while upregulating eNOS mRNA expression, further supporting its protective effects on endothelial function (Xia et al. 2014).

Zedoarondiol (ZAD), a sesquiterpene lactone, enhances ox‐LDL‐induced SOD activity in ECs, reduces ROS and malondialdehyde (MDA) levels, improves cell survival, and inhibits lactate dehydrogenase (LDH) activity. Additionally, ZAD reduces the release of inflammatory factors such as IL‐1*β*, TNF‐*α*, and MCP‐1. This protective mechanism is attributed to the deregulation of Keap1 and LOX‐1 expression, alongside Nrf2 nuclear translocation and upregulation of NQO1 and HO‐1 expression (Mao et al. 2018).

Lycopene, a carotenoid found in tomatoes, exhibits antioxidant, anti‐tumor, and cardiovascular protective properties (Ali et al. 2020; Bin‐Jumah et al. 2022; Caseiro et al. 2020; Ranjan et al. 2019). Lycopene acts as a hypotensive agent by inhibiting angiotensin‐converting enzyme (ACE) and modulating NO bioavailability. It plays a key role in preventing atherosclerosis by lowering LDL cholesterol levels and increasing HDL levels (Bin‐Jumah et al. 2022). Lycopene also inhibits cyclic strain‐induced ET‐1 expression in ECs by suppressing ROS production, thereby halting the progression of atherosclerosis (Sung et al. 2015).

Parthenolide, a sesquiterpene lactone extracted from wild chamomile and other plants, has demonstrated significant anti‐tumor, antioxidant, and antibacterial activities. Animal studies have shown that parthenolide reduces oxidative stress and downregulates caspase‐1 mRNA expression, thus mitigating the progression of atherosclerosis (Sultan et al. 2023).

Hydroxytyrosol, a polyphenolic compound found primarily in olives, possesses strong antioxidant, anti‐inflammatory, and anti‐atherosclerotic effects (Vijakumaran et al. 2023). Its anti‐inflammatory mechanism is thought to involve inhibition of the NF‐κB pathway in VECs. Additionally, the ability of hydroxytyrosol to reduce ROS formation in ECs suggests its potential for treating atherosclerosis (Marrero et al. 2023).

### 4.5 Alkaloid

Plant alkaloids are significant natural compounds with medicinal properties, widely utilized in healthcare and disease prevention. These bioactive molecules are prevalent throughout the natural world, and human research on alkaloids has a long history. Alkaloids continue to serve as vital resources for identifying potential drug candidates. Some alkaloids have been shown to reduce oxidative stress in vivo by activating the Nrf2 pathway, stimulate the Akt signaling pathway, regulate lipid metabolism in VECs, and mitigate vascular damage caused by apoptosis. It has been observed that the alkaloids corynoline, oxymatrine, and berberine exert their respective biological activities predominantly by modulating the expression levels of HO‐1 and Nrf2 (Song et al. 2020; Wu et al. 2019; Zhang, Lang, et al. 2023).

In addition, certain alkaloids inhibit the production of inflammatory factors, suppress the activity of pathways such as NF‐κB and TLR4, and modulate the expression of non‐coding RNAs, thereby exhibiting anti‐inflammatory effects.

Colchicine, an alkaloid derived from plants of the *Colchicum* genus, has been traditionally used in China to alleviate swelling and pain. Its therapeutic application, especially in the treatment of gout, is well documented (Stamp et al. 2025). Beyond gout, colchicine has also been employed in treating other conditions, including coronary artery disease and various inflammatory disorders (Zhang, Weber, et al. 2024). Although the pharmacological mechanisms of colchicine in different diseases are not fully elucidated, studies suggest that low‐dose colchicine significantly inhibits atherogenesis.

Oxymatrine (OMT), an alkaloid extracted from the legume *Sophora japonica* , has been shown in mouse studies to effectively reduce fat accumulation and, consequently, the formation of atherosclerotic plaques. Homocysteine (Hcy), a sulfur‐containing amino acid, is strongly associated with cardiovascular disease and is considered a major risk factor (Zhang, Ren, et al. 2016). Hcy contributes to endothelial damage through a series of complex mechanisms that drive atherosclerosis development, ultimately leading to cardiovascular disease. Studies indicate that Hcy is highly cytotoxic, inducing EC apoptosis. It also elevates the levels of ROS, MDA, and LDH, while reducing those of SOD in ECs, thus exacerbating atherosclerosis progression. Research has demonstrated that OMT protects ECs from Hcy‐induced damage by inhibiting MMPs expression, increasing the Bcl‐2/Bax ratio, and suppressing the expression of caspase‐3 and caspase‐9. Additionally, OMT enhances the phosphorylation of eNOS and Akt (Wu et al. 2019).

Corynoline, an alkaloid derived from Corydalis species, exhibits potential anti‐inflammatory properties. One study investigated the mechanism underlying corynoline's anti‐inflammatory effects in LPS‐stimulated ECs. The findings revealed that corynoline markedly inhibited the LPS‐induced expression of VCAM‐1, ICAM‐1, IL‐8, and TNF‐*α* (Liu et al. 2018). To further elucidate the mechanism, researchers examined the activation of NF‐κB and the expression of Nrf2. The results demonstrated that corynoline suppressed LPS‐induced NF‐κB activation while upregulating Nrf2 and HO‐1. Notably, knockdown of Nrf2 reversed corynoline's anti‐inflammatory effects (Wang, Luo, et al. 2022; Zhang, Lang, et al. 2023). Corynoline also reduced the production of TNF‐*α* and IL‐8 in ECs and lowered the expression of ICAM‐1 and VCAM‐1. Additionally, it inhibited LPS‐induced phosphorylation of IκB*α* and NF‐κB p65 (Zhang, Lang, et al. 2023).

BBR is a versatile alkaloid extracted from Rhizoma Coptidis with a wide range of biological activities, including anti‐inflammatory, antitumor, antidiabetic, and antibacterial effects (Chen et al. 2025). Studies have demonstrated that BBR alleviates H_2_O_2_‐induced EC damage, inhibits ROS production and apoptosis (Song et al. 2020). Therefore, the potential inhibitory effects of Nrf2‐siRNA should be considered when using BBR therapeutically. Berberine has also been shown to significantly lower serum cholesterol levels in mice by inducing the expression of LDL receptors in hepatocytes. A study investigating the effects of berberine on atherosclerosis in apolipoprotein E‐deficient (ApoE^−/−^) mice found that berberine enhanced the uptake of modified LDL (DiO‐Ac‐LDL) by inducing macrophage scavenger receptor A (SR‐A) expression. This upregulation of SR‐A was also observed in vivo in macrophage foam cells and at atherosclerosis lesion sites. In RAW264.7 cell experiments, berberine induced SR‐A expression by inhibiting PTEN expression, leading to sustained activation of the Akt signaling pathway, promotion of cellular metabolism, and maintenance of normal cell survival and growth. This mechanism contributed to the inhibition of atherosclerosis progression (Li et al. 2009).

Liensinine, a bisbenzylisoquinoline alkaloid primarily found in the seeds of lotus ( *Nelumbo nucifera* Gaertn.), exhibits both anti‐inflammatory and antitumor properties (Wang, Ma, et al. 2024). Research indicates that liensinine mitigates vascular inflammation by inhibiting inflammatory mediators in macrophages and targeting SMCs to regulate their proliferation and migration. Additionally, *Lotus corniculatus* has demonstrated DPPH free radical scavenging activity in vitro, along with reducing NO production in RAW 264.7 cells. *Lotus corniculatus* also inhibits PDGF‐stimulated SMC proliferation. Collectively, these findings suggest a potential preventive effect of rosmarinine on vascular inflammation, indicating its promise as a therapeutic agent for managing inflammation (Jun et al. 2021).

Sinapine, an alkaloid isolated from the seeds of cruciferous plants, has demonstrated anti‐inflammatory and antioxidant properties, effectively reducing oxidative stress and thereby inhibiting the progression of atherosclerotic plaques (Chadni et al. 2023).

Sinigrin, the principal mustard glucosinolate found in cruciferous plants, possesses potent antioxidant, antitumor, and anti‐inflammatory effects. Animal studies have shown that sinigrin suppresses oxidative stress in cerebral ischemia–reperfusion injury by inhibiting the TLR4/MyD88 signaling pathway, which in turn helps prevent or slow the progression of atherosclerosis (Guo et al. 2024).

Capsaicin, a natural compound extracted from chili peppers, has been shown to significantly upregulate the expression of miR‐126, which plays a protective role in the vascular endothelium. This regulation helps to repair endothelial damage and inhibits the progression of atherosclerosis (Ates et al. 2022).

Evodiamine, an alkaloid found in the fruit of *Cornus officinalis* , possesses antioxidant and anti‐inflammatory properties. Research indicates that evodiamine reduces inflammation in mice by inhibiting the phosphorylation of AKT, NF‐κB p65, ERK1/2, p38, and JNK, which in turn slows apoptosis. Furthermore, Wuzhuganine has been found to significantly improve lipid metabolism in mice by reducing blood levels of triglycerides (TG), total cholesterol (TC), and LDL‐C. This suggests that Wuzhuganine may inhibit atherosclerosis plaque formation and reduce EC apoptosis (Wei et al. 2023).

### 4.6 Stilbene

Astragaloids (stilbene compounds) refer to a class of substances characterized by homodiphenylstilbene parent nuclei or their polymers. These compounds are present in low concentrations in the normal tissues of plants, with mono‐ and di‐phenolic hydroxystilbene compounds predominantly located in the thin‐walled cells of the plant xylem. Notably, the levels of astragaloids in plants increase sharply when plant tissues are damaged or exposed to external stimuli, suggesting that natural astragaloids may act as stress response compounds in certain plants (Dubrovina and Kiselev 2017). Most astragaloids exhibit potent antioxidant activity, primarily through mechanisms such as upregulating Nrf2, PPAR‐*α*, and other signaling pathways to reduce ROS levels, thereby exerting their antioxidant effects. Additionally, some astragaloids inhibit the expression of pro‐inflammatory factors such as TNF‐*α* and IL‐1*β*, thereby reducing inflammation.

Pterostilbene (PT), a stilbene analogue, exhibits notable anti‐inflammatory and anti‐tumor properties. It has been shown to mitigate the inhibitory effects of uremic serum on EC proliferation. The underlying mechanism involves PT restoring the activities of SOD and CAT, which are reduced by uremic serum, while also preventing increases in MDA, superoxide anion levels, H_2_O_2_, and NAD(P)H oxidase activity in ECs. By alleviating intracellular oxidative stress, PT reverses the US‐induced inhibition of EC proliferation. Additionally, PT significantly suppresses the expression of pro‐inflammatory cytokines, including TNF‐*α*, IL‐1*β*, VCAM‐1, MCP‐1, and iNOS (Stewart et al. 2010). Further research has demonstrated that PT downregulates Keap1 levels in a dose‐dependent manner, while simultaneously highlighting its potential as an anti‐atherosclerotic agent with antioxidant, anti‐inflammatory, and anti‐cytotoxic properties to protect against US‐mediated endothelial damage (Dubrovina and Kiselev 2017). In animal studies, pretreatment with PT effectively reduced lipid accumulation and significantly enhanced the expression of Nrf2, PPAR‐*α*, HO‐1, and AMPK in mice (Shen et al. 2023). Moreover, PT was found to reduce aortic plaque size and macrophage infiltration in rats on a high‐fat diet, and it inhibited oxidative stress and H_2_O_2_‐induced EC apoptosis in vitro. These protective effects are largely attributed to PT's modulation of the Nrf2 pathway (Pan et al. 2018).

Resveratrol (RES), a non‐flavonoid polyphenolic compound, is a phytoalexin produced by various plants in response to stress and can be synthesized in grape leaves and skins. It is a biologically active component found in wine and grape juice, with a broad range of biological activities, including antimicrobial, antioxidant, anti‐aging, cardiovascular protective, and anti‐tumor effects (Meng et al. 2020; Sharifi‐Rad et al. 2022; Xia et al. 2017). RES is a naturally occurring antioxidant that has garnered significant attention for its potential health benefits, such as anticancer, anti‐aging, and antimicrobial properties. It is well tolerated in humans and has recently been used as a health supplement (Ren et al. 2021; Zhang et al. 2008).

Research indicates that RES attenuates inflammation in rats primarily through the PI3K‐Akt‐mTOR signaling pathway (Feng et al. 2022). Moreover, RES has been shown to reduce ROS production in ECs triggered by PA, significantly lowering ROS levels (Song et al. 2018). In addition, RES promotes autophagy in ECs and mitigates PA‐induced intracellular ROS through autophagy regulation via the AMP‐activated protein kinase (AMPK)‐mTOR pathway (Yamamoto et al. 2010). Furthermore, studies have demonstrated that RES reduces neutrophil extracellular trap (NET) release and significantly decreases H_2_O_2_ levels in activated neutrophils (de Mattos et al. 2024). In animal models, RES has shown positive effects on atherosclerosis, ischemic heart disease, arrhythmia, and heart failure. Although the precise mechanisms remain unclear, some cardiovascular benefits of RES are thought to be mediated through the activation of SIRT1, AMPK, and endogenous antioxidant enzymes.

Piceatannol is a compound derived from astragaloids, found in significant quantities in the seeds of passion fruit, with demonstrated antioxidant and anti‐tumor properties (Hosoda et al. 2021). Animal studies have shown that long‐term administration of piceatannol in rats can markedly enhance vascular relaxation and prevent vascular sclerosis. Additionally, cellular experiments indicate that piceatannol upregulates both eNOS mRNA and eNOS protein expression, suggesting that it may contribute to improving human vascular health through this mechanism (Kinoshita et al. 2013).

### 4.7 Quinone

Quinone compounds are prevalent in various plants, particularly in species such as rhubarb, Heshouwu ( *Polygonum multiflorum* ), and tiger nuts, where they are found in high concentrations. Several quinones exhibit antioxidant properties, regulate lipid metabolism, and inhibit platelet aggregation. Some quinones have been shown to activate the Nrf2 signaling pathway and modulate the Rho kinase system, thereby reducing oxidative stress in vivo and protecting cardiovascular ES from H_2_O_2_‐induced damage.

Miltirone, a phenanthrenequinone compound derived from *Salvia miltiorrhiza* , possesses both anti‐tumor and cardiovascular protective properties. It has been demonstrated to significantly improve the survival of ox‐LDL‐induced EA.hy926 cells and inhibit intracellular ROS formation (Akaberi et al. 2016). Furthermore, miltirone alleviates ox‐LDL‐induced EC injury by activating the Nrf2/HO‐1 signaling pathway. The JNK pathway is also involved in this Nrf2/HO‐1 activation, suggesting that miltirone may help ameliorate atherosclerosis (Zhang, Zhang, et al. 2016). In addition to its effects on endothelial protection, miltirone inhibits platelet aggregation in a dose‐dependent manner by suppressing the release of dense and *α*‐granules. It also reduces platelet clot contraction and expansion by inhibiting phosphorylation of PLCγ2, PKC, Akt, GSK3*β*, and ERK1/2 in the downstream signaling pathways of platelet collagen receptors. Moreover, miltirone reduces phosphorylation of Src and FAK, though it does not affect *β*3 in integrin *α*IIb*β*3‐mediated outside‐in signaling. These findings suggest that miltirone may be a promising candidate for preventing atherosclerosis by inhibiting platelet aggregation (Song et al. 2019).

Tanshinone IIA exhibits a wide spectrum of biological activities, including vasodilation, anti‐atherosclerotic, antioxidant, anti‐inflammatory, anti‐tumor, antihypertensive, and anti‐adipogenic effects (Li and Wang 2016; Li et al. 2016). Increasing evidence suggests that this compound has a protective effect on ECs under conditions of fluctuating hyperglycemia. The underlying mechanism is thought to involve reducing oxidative stress, decreasing lipid peroxidation and MDA levels, enhancing antioxidant enzyme activity, and promoting NO release through modulation of the Rho kinase system (Ansari et al. 2021). In addition, tanshinone IIA has been shown to reduce atherosclerotic plaque formation without significantly altering lipid profiles (He et al. 2023). In addition, tanshinone IIA has been shown to reduce atherosclerotic plaque formation without significantly altering lipid profiles (Tang et al. 2011). It also increases collagen content in atherosclerotic plaques, likely through its anti‐inflammatory effects and inhibition of MMP‐1 expression.

Rhein, an anthraquinone, possesses anti‐inflammatory, antioxidant, and anticancer properties (Li, Chen, et al. 2024; Liu, Shu, et al. 2023). Research indicates that rhein may prevent atherosclerosis by downregulating the expression of caspase‐3, ‐8, ‐9, and BID mRNAs, and by protecting ECs from H_2_O_2_‐induced damage. These protective effects of rhein have been demonstrated in various studies (Zhong et al. 2012).

Shikonin, the primary active ingredient in *Lithospermum erythrorhizon* (comfrey), has strong antioxidant, antibacterial, and anti‐inflammatory activities. Studies have shown that shikonin effectively inhibits H_2_O_2_‐induced expression of caspase‐3 and caspase‐9, increases Bcl‐2 expression, and reduces the expression of Bax and cytochrome c in HT29 cells. By eliminating ROS, improving mitochondrial function, and attenuating H_2_O_2_‐induced oxidative damage, shikonin prevents apoptosis (Zhong et al. 2021).

### 4.8 Others With Evidence From Clinical Trials

Artesunate is a derivative from artemisinin from the traditional Chinese herb sweet wormwood. Jiang et al. induced an atherosclerosis model in AOPE^−/−^ mice by a Western diet, demonstrating that the combination of rosuvastatin and artesunate was more effective in attenuating the progression of atherosclerotic lesions compared to monotherapy with either agent, suggesting that the co‐administration of lipid‐lowering and anti‐inflammatory therapies may confer enhanced cardiovascular protection (Jiang et al. 2016).

In an observational study involving 624 participants, the intake of anthocyanin‐rich products was found to be associated with specific plasma metabolites, as determined through regression analysis based on the results of targeted metabolomic profiling (Mostafa et al. 2023). Chios Mastiha essential oil (CMO) is a natural product extracted from the resin of Mastiha. In another study, 192 healthy volunteers were enrolled, among whom 160 exhibited total cholesterol levels exceeding 200 mg/dL. Following 8 weeks of CMO administration, significant reductions in total cholesterol and low‐density lipoprotein cholesterol (LDL‐C) were observed in the CMO‐treated group compared to the placebo group (Kartalis et al. 2024).

In another study, 72 volunteers underwent an 8‐week intervention with a fermented product primarily derived from Chenpi (dried citrus peel). Compared to the control group, the intervention led to significant reductions in body weight and serum triglyceride levels. Moreover, the product enhanced specific gut microbial taxa associated with lipid metabolism regulation (Tan et al. 2025).

In summary, various types of natural products exert anti‐atherosclerotic effects through multiple mechanisms, primarily focusing on the inhibition of oxidative damage and inflammatory responses. However, most reports on natural products are based on cellular and animal models, with limited clinical studies. Future research should focus on designing more scientifically rigorous clinical safety and efficacy trials, as well as modifying natural compounds to reduce their toxicity and improve their bioavailability, thereby driving the development of this industry.

## 5 Proprietary Chinese Medicines for Cardiovascular Disease Treatment

A proprietary Chinese medicine refers to any proprietary product composed solely of TCM as the active ingredient and formulated in a finished dose form (e.g., tablets, capsules, pills, etc.). Based on TCM literature, clinical validation, and modern pharmaceutical technology, several proprietary Chinese medicines have been developed for treating atherosclerosis (Table 3).

**TABLE 3:** Summary of proprietary Chinese medicines for cardiovascular disease treatment.

PCM	Model	Mechanism of action	References
Xuezhikang tablets	TNF‐*α*‐induced HAECs and High‐fat chow‐induced SD rats.	Anti‐inflammatory (through downregulation of IL‐6 and TNF‐α).	Lin et al. (2011); Zhu et al. (2013)
Xuesaitong tablets	ox‐LDL‐induced A7r5 cells.	Anti‐inflammatory (through downregulation of IL‐6 and TNF‐α).	Zhao et al. (2018)
Fufang Danshen Dripping Pills	ox‐LDL‐induced VSMCs.	Antioxidant (via intracellular ROS scavenging).	Li et al. (2022)
Wenxin Granules	Isoproterenol‐induced SD rats.	Alleviates myocardial fibrosis (via inhibition of *β*1‐AR/TGF‐*β*1/Smad2 signaling).	Wang et al. (2023)
Shexiang Baoxin pills	MI/R post‐surgical C57BL/6 mice.	Slowing myocardial injury (via enhanced angiogenesis).	Xu et al. (2024)
Fufang Sishen granules	High‐fat chow‐induced guinea pigs	Reduces myocardial injury (via upregulation of Bcl‐2 and downregulation of Bax/Fas).	Xia et al. (2017)

Xuezhikang tablets are included in the Chinese Pharmacopoeia as an adjunct therapy for hyperlipidemia and cardiovascular diseases associated with atherosclerosis. The primary active component, monacolin K, is well‐known for its cholesterol‐lowering properties (Lin et al. 2011; Zhu et al. 2013). Shi et al. performed a network meta‐analysis and found that Xuezhikang effectively increased serum HDL‐C levels in patients with hyperlipidemia, thereby contributing to the maintenance of normal cholesterol metabolism and exhibiting potential anti‐atherosclerotic activity (Shi et al. 2025).

Xuesaitong tablets (XST) promote blood circulation, remove blood stasis, and inhibit platelet aggregation. They are rich in *Panax ginseng* total saponins, which have been shown to inhibit the progression of atherosclerosis through antioxidant mechanisms and platelet aggregation inhibition. Therefore, it is inferred that Xuesaitong tablets may be effective in treating or supporting the treatment of atherosclerosis (Zhao et al. 2018). Gao et al. (2022) performed a systematic review with meta‐analysis and found that XST can significantly improve plasma viscosity, whole‐blood viscosity at high and low shear rates, fibrinogen, and hematocrit, indicating its potential anti‐atherosclerotic effects.

Fufang Danshen Dripping Pills consist of *Salvia miltiorrhiza* , *Panax ginseng*, and other medicinal herbs that activate blood circulation, remove blood stasis, regulate qi, and relieve pain. *Salvia miltiorrhiza* contains tanshinone and tanshinone IIA, which can reduce oxidative stress in vascular ECs and inhibit inflammation by regulating multiple signaling pathways. *Panax ginseng* is rich in ginsenosides, which inhibit platelet aggregation. Therefore, Fufang Danshen pills may help reduce or reverse atherosclerosis (Li et al. 2022). The systematic review with meta‐analysis by Zhang et al. demonstrated that Fufang Danshen injection can effectively reduce blood lipid levels in patients with hyperlipidemia, thereby contributing to cardiovascular protection and exerting anti‐atherosclerotic effects (Zhang, Yang, Pang, et al. 2024).

Wenxin granules, composed primarily of *Codonopsis*, *Rhizoma polygonati*, *Panax ginseng*, and Succinum, are indicated for coronary heart disease. These granules benefit qi, nourish yin, improve blood circulation, and remove blood stasis. *Codonopsis* is rich in alkaloids, flavonoids, terpenoids, and other compounds, with flavonoids being the main active ingredients, offering antioxidant and anti‐inflammatory benefits (Wang et al. 2023). A clinical trial showed that the combination of Wenxin Granules and aspirin was more effective than aspirin monotherapy in reducing levels of endothelin‐1 and high‐sensitivity C‐reactive protein, as well as in increasing nitric oxide (NO) levels. These effects contributed to the suppression of systemic inflammation and endothelial dysfunction, suggesting anti‐atherosclerotic properties of Wenxin Granules (Cao et al. 2021).

Tongxinluo capsules are primarily composed of traditional Chinese medicinal herbs, including Ginseng, Scorpion, Red Peony Root, Centipede, Sandalwood, and Dalbergia. These components work synergistically to invigorate Qi, promote blood circulation, and alleviate pain by unblocking meridians. In an in vitro study, Jiang et al. demonstrated that Tongxinluo effectively inhibits ox‐LDL‐induced oxidative damage in mouse aortic endothelial cells (MAECs), primarily by reducing intracellular ROS levels (Jiang et al. 2023). Using an atherosclerotic rabbit model, Qi et al. demonstrated that TXL effectively inhibits the progression of atherosclerosis in New Zealand White rabbits. The underlying mechanism is primarily attributed to the attenuation of systemic inflammatory responses and the enhancement of plaque stability, which may be mediated through the modulation of gut microbiota composition (Qi et al. 2022). In a clinical study involving 324 volunteers, it was demonstrated that the combination therapy of Tongxinluo and atorvastatin is more effective than conventional treatment in dissolving or inhibiting atherosclerotic plaques. Moreover, this combination exhibited a favorable safety profile, with no adverse events reported during the study (Wang et al. 2019).

Shexiang Baoxin pills are composed of artificial musk, ginseng extract, artificial bezoar, cinnamon, Suhexiang (styrax), toad venom, and borneol, among other ingredients. Artificial musk promotes blood circulation and reduces blood stasis, while ginseng replenishes qi and blood, enhancing circulation. Cinnamon has warming properties that invigorate yang and open blood vessels; Suhexiang provides analgesic effects through its mild aroma, and toad venom eliminates dampness and alleviates pain. Artificial bezoar clears heat, removes phlegm, and restores consciousness, while borneol alleviates depression and relieves pain (Wang et al. 2023). Ginseng extract is rich in ginsenosides, such as ginsenoside Ra1 and ginsenoside Rb1, which effectively inhibit the progression of atherosclerosis (Hou et al. 2024). Cinnamon contains cinnamic acid, which inhibits apoptosis in cardiomyocytes, and artificial musk is abundant in peptides, proteins, and other bioactive macromolecules with potent anti‐inflammatory properties (Lv et al. 2022). Liu et al. demonstrated that cinnamon powder significantly reduced blood glucose levels in diabetic BALB/c mice induced by a high‐fat diet and streptozotocin (HFD/STZ). Their findings indicated that cinnamon powder modulates glucose metabolism by activating the PI3K/AKT and AMPK*α*/PGC1*α* signaling pathways, thereby suppressing hepatic gluconeogenesis and enhancing hepatic glycogen synthesis in diabetic mice (Liu, Liu, et al. 2023). In addition, one study confirmed that the ethanol extract of cinnamon could effectively reduce the expression of malondialdehyde (MAD) and improve dyslipidemia in rats (Dewi Ratih et al. 2023). A study using a high‐fat diet‐induced rat model of hepatic steatosis and oxidative injury demonstrated that cinnamon powder effectively attenuated hepatic lipid accumulation and oxidative damage. These protective effects were primarily mediated through the upregulation of PPAR*α*, CD36, CPT‐1*β*, and FAS expression, indicating that cinnamon and its derivatives possess bioactive properties relevant to the prevention and treatment of atherosclerosis (Li et al. 2022). Cinnamaldehyde, the active compound in cinnamon, can effectively inhibit the further development of atherosclerosis in high‐fat diet‐induced ApoE^−/−^ mice through the IκB/NF‐κb signaling pathway (Li et al. 2019).

Shexiang Baoxin pills have been found to elevate serum levels of 20‐HETE (20‐hydroxyeicosatetraenoic acid) in rats with myocardial infarction (MI), an effect that can be inhibited by HET0016, a selective inhibitor of the enzyme responsible for 20‐HETE synthesis. This suggests that Shexiang Baoxin pills may exert their therapeutic effects by modulating the 20‐HETE pathway (Huang et al. 2017). These changes in biomarkers contributed to the inhibition of inflammation and EC oxidative damage, suggesting that Shexiang Baoxin pills may possess potential anti‐atherosclerotic effects.

Naoxintong capsules are primarily composed of traditional Chinese medicinal herbs, including *Angelica sinensis* (Danggui), *Semen persicae* (Taoren), and *Carthami flos* (Honghua). Wan et al. found that Naoxintong effectively inhibits the early development of atherosclerosis in mice, primarily through modulation of the gut microbiota (Wan et al. 2024). In a systematic review and meta‐analysis of randomized trials, Liang et al. concluded that Naoxintong is an effective and safe therapeutic option for the treatment of atherosclerosis (Liang et al. 2018).

Shen et al. demonstrated, through a clinical trial, that Dengzhan Shengmai capsule has comparable efficacy to aspirin in the treatment of carotid atherosclerotic plaques and exhibits a more favorable safety profile. This therapeutic approach may offer a promising alternative strategy, particularly for patients with aspirin intolerance (Shen et al. 2022).

In summary, a substantial body of evidence from cellular, animal, and clinical studies has demonstrated that various Chinese patent medicines possess direct or indirect anti‐atherosclerotic activity. Despite the abundant evidence on the potential, issues still persist, including the limited clinical validation and weak therapeutic effects when used alone. Future research should focus on more rigorously designed safety and efficacy trials based on a large sample size.

## 6 Discussion

This review examines TCM and natural product monomers identified in recent years for their protective effects against atherosclerosis, where the prevention of oxidative stress‐induced damage to ECs is an important aspect. The main bioactive substances include flavonoids, phenylpropanoids, alkaloids, terpenoids, astragaloids, and quinones. Many of these compounds have been shown to mitigate EC apoptosis induced by ox‐LDL, H_2_O_2_, Hcy, and high glucose‐induced oxidative stress. The activation of the Nrf2/HO‐1 signaling pathway is the predominant mechanism (Figure 5). These results elucidate the mechanisms of TCM products and provide reference for the standardization of TCM, while the interaction between different components deserves further investigation.

**FIGURE 6:**
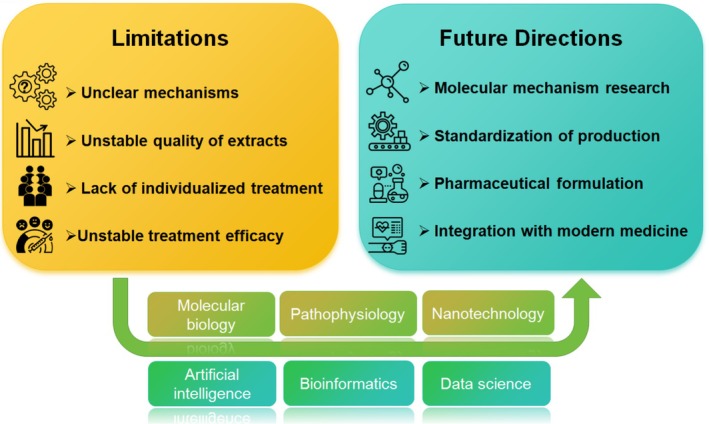
Limitations and future directions of the research of TCM in the treatment of atherosclerosis.

Despite the promising anti‐atherogenic effects of TCM components, several critical questions remain unanswered (Figure 6). Most of the natural products discussed in this review have only been evaluated in preliminary pharmacological studies, and the molecular mechanisms underlying their anti‐atherogenic activity are still poorly understood. Research on the mechanisms of action of TCM formulas is often speculative, with much of the analysis relying on the active compounds found in their key ingredients. The reductionist approach of studying isolated compounds (e.g., ginsenosides, tanshinones), while mechanistically informative, may overlook the synergies inherent in TCM formulations. Future studies should integrate quantitative systems pharmacology to decode the multi‐target, multi‐component nature of TCM. For example, Fufang Danshen Dripping Pills have been clinically shown to be very effective, but the independent active ingredients have not yet been elucidated, and it is only assumed that the tanshinone and ginsenosides in them work through anti‐inflammation and inhibition of platelet aggregation (Li et al. 2022). Active compounds of TCMs are known to inhibit the production of pro‐inflammatory factors and subsequently block key signaling pathways, reducing inflammation. Meanwhile, some compounds can regulate non‐coding RNAs, inhibiting atherosclerosis progression through post‐transcriptional regulation. Given the complexity of the active ingredients in TCM, isolating and studying individual components pose significant challenges. Additionally, there is a scarcity of pharmacokinetic (PK) data and clinical studies on these natural extracts or monomers, and investigations into their toxicity and organ‐specific effects remain insufficient. For instance, despite growing evidence of the protective effects of natural products on ECs through Nrf2/HO‐1 activation, more comprehensive preclinical studies are necessary to further develop new drugs for atherosclerosis and other cardiovascular diseases. The quality of herbal medicine depends on many factors, including the place of origin, cultivation methods, preservation conditions, and so forth, where standardized quality control is still an unmet challenge due to the complexity of bioactive components. These unmet challenges lead to the instability of product quality and treatment efficacy, limiting the ability of individualization in treating atherosclerosis, which is a core principle of TCM.

To ensure the stability and consistency of active ingredients in TCM formulas, modern analytical techniques such as high‐performance liquid chromatography–tandem mass spectrometry (HPLC–MS/MS) enable precise quantification of marker compounds and detection of batch‐to‐batch variations. Fingerprint chromatography combined with chemometric analysis provides comprehensive quality assessment by establishing characteristic chemical profiles for reference standardization. Additionally, real‐time process analytical technology (PAT) and near‐infrared spectroscopy (NIRS) allow for dynamic monitoring of critical quality attributes during production to maintain consistent therapeutic efficacy.

Although several marketed TCM compound formulations have demonstrated satisfactory therapeutic efficacy, their complex composition, unique theoretical foundation, as well as the dependence on the expertise of practitioners present distinct challenges. Furthermore, given that TCMs are derived from natural sources with chemically diverse constituents, their quality is significantly influenced by external factors including geographical origin, soil composition, climate, and cultivation techniques. Additionally, the intricate processing procedures further complicate quality control, rendering it a major hurdle in the standardization and modernization of TCM products (Zhang, Zhang, et al. 2022). In light of the current challenges in quality control, the concept of quality markers (Q‐markers) has been introduced in TCM. Q‐markers refer to inherent or process‐generated compounds present in raw herbal materials or final TCM products. These substances are closely associated with the pharmacological characteristics of TCM, serving as indicators of both efficacy and safety, and thus represent a scientific basis for the standardized evaluation and quality control of TCM formulations (Mu et al. 2025). Chemical fingerprinting technology has been applied in the prediction of Q‐markers from a fingerprint recognition perspective, where spectroscopic analytical methods often play a pivotal role. Currently, NIRS, liquid chromatography–tandem mass spectrometry (LC–MS/MS), and liquid chromatography–mass spectrometry (LC–MS) are the most commonly employed techniques. Due to its distinct advantages in rapid and non‐destructive detection, NIRS has emerged as one of the most extensively used technologies in the quality control of TCM. As a representative spectroscopic technique, NIRS offers advantages such as speed, simplicity, and cost‐effectiveness, and is capable of acquiring spectral data from both solid and liquid samples, thereby enabling quantitative analysis of various components within complex TCM matrices (Yang et al. 2024).

Clinical trials play a key role in validating the efficacy of natural products and plant‐derived compounds. Currently, most clinical trials are single‐center and small‐size ones (Table 4). Consequently, statin‐based pharmacotherapy remains the predominant approach in clinical practice (Sever et al. 2025).

**TABLE 4:** Mechanistic classification of natural products and TCMs in AS treatment.

Object	Mechanistic classification	References
Anti‐inflammatory mechanisms		
*Panax notoginseng* (Burk.) F. H. Chen; *Erigeron breviscapus* (Vant.) Hand.‐Mazz.; Phenolic acids; Flavonoids; Alkaloids; Xuesaitong tablets Shexiang Baoxin pills	NF‐κB inhibition	Wang et al. (2011); Li et al. (2020); Jo et al. (2024); Liu, Zhang, et al. (2023); Wang, Luo, et al. (2022); Zhao et al. (2018)
*Ginkgo biloba* L.	PI3K/AKT regulation	Pu et al. (2024)
Flavonoids	TLR4/NF‐κB/NLRP3 inhibition	Syeda et al. (2019); Li et al. (2020)
Phenylpropanoids	Activation of MMP‐2 and MMP‐9	Liu, Yan, et al. (2021)
Antioxidant mechanisms		
*Arctium lappa* L.; *Astragalus membranaceus* (Fisch.) Bunge; *Ganoderma tsugae* Murr.; *Prunella vulgaris* L.; Phenylpropanoids; Flavonoids; Phenolic acids; Terpenoids; Stilbene; Quinone	Nrf2/HO‐1/eNOS activation	Ruan et al. (2022); Yang et al. (2016); Wei et al. (2009); Hwang et al. (2012); Liu, Yan, et al. (2021); Syeda et al. (2019); Li et al. (2020); Zhou et al. (2009); Wang, Luo, et al. (2022); Pan et al. (2018); Zhang, Zhang, et al. (2016)
*Panax ginseng* C.A. Mey; *Ginkgo biloba* L.; *Ganoderma tsugae* Murr.	Modulating SOD, GSH activity	Zhang, Yu, et al. (2023); Guo et al. (2021); Chen et al. (2024)
Terpenoids; Phenylpropanoid	Scavenging ROS	Jiang et al. (2015); Chniguir et al. (2024)
Improvement of fat metabolism		
*Arctium lappa* L.	Activates AMPK/ACC/CPT‐1	Ma et al. (2022)
Xuezhikang tablets	Maintain cholesterol metabolic homeostasis	Zhu et al. (2013)
Naoxintong capsules	Reduce the patient's blood lipid levels	Wan et al. (2024); Liang et al. (2018)
Chenpi Jiaosu	Maintaining normal lipid levels	Tan et al. (2025)

Future research should prioritize investigating the pharmacokinetics, systemic side effects, and toxicity of these drug candidates. On the one hand, further investigation on the molecular mechanisms is necessary, where network pharmacology based on the latest data science, artificial intelligence, and bioinformatics can be an efficient tool. On the other hand, clinical studies are needed to comprehensively evaluate the treatment efficacy. Cardiovascular diseases often require prolonged treatment, and oral administration is the preferred route for managing atherosclerosis and other cardiovascular diseases. However, many of the compounds reviewed, such as those derived from *Lotus corniculatus* and curcumin, exhibit low bioavailability when taken orally. Therefore, it is crucial to develop appropriate pharmaceutical formulations for these drug candidates under the framework of modern medicine. In addition, modifying existing monomer compounds to reduce toxicity and increase solubility could improve their efficacy and enhance human tolerance for long‐term use. The standardization of production can improve the quality of TCM products and plays a key role in pharmaceutical formulation. To address the current research challenges, it is essential to develop a multidisciplinary approach (Figure 6) including molecular biology, biochemistry, pathophysiology, nanotechnology, artificial intelligence, data science, and bioinformatics with network pharmacology, towards the modernization of TCM‐based treatment of atherosclerosis.

## 7 Conclusion

TCM and its modern products have shown potentials in treating atherosclerosis. The possible mechanisms include regulation of blood lipids, anti‐lipid peroxidation, anti‐polymerization, anti‐coagulation, and pro‐fibrinolysis, inhibition of smooth muscle cell proliferation, and protection of vascular endothelial function.

Despite the potentials and increasing number of bioactive components being identified, there are unmet challenges in mechanism research, quality control, and standardization, which call for multidisciplinary collaboration for future research. Current assessments of herbal medicine safety and efficacy continue to encounter significant methodological limitations, particularly regarding standardization and study design consistency. Although certain botanicals demonstrate clinically relevant anti‐atherosclerotic effects, their therapeutic application necessitates expert oversight to mitigate risks associated with herb‐drug interactions and batch‐to‐batch variability. To advance the field, subsequent research must implement pharmacologically standardized preparations, robust quality assurance protocols, and methodologically sound clinical trials to generate reproducible evidence regarding both therapeutic potential and safety considerations.

## Author Contributions

**Dilaram Nijat:** conceptualization, methodology, software, writing – original draft, writing – review and editing. **Qingzhe Zhao:** data curation, writing – original draft, software. **Gulhasal Abdurixit:** visualization, investigation. **Jianhua He:** software, validation. **Haipeng Liu:** visualization, conceptualization, supervision, writing – original draft, writing – review and editing. **Jinyao Li:** funding acquisition, investigation, validation.

## Conflicts of Interest

The authors declare no conflicts of interest.

## Data Availability

Data sharing is not applicable to this article as no datasets were generated or analysed during the current study.
